# Three-dimensional-printed individualized porous implants: A new “implant-bone” interface fusion concept for large bone defect treatment

**DOI:** 10.1016/j.bioactmat.2021.03.030

**Published:** 2021-04-06

**Authors:** Teng Zhang, Qingguang Wei, Hua Zhou, Zehao Jing, Xiaoguang Liu, Yufeng Zheng, Hong Cai, Feng Wei, Liang Jiang, Miao Yu, Yan Cheng, Daoyang Fan, Wenhao Zhou, Xinhong Lin, Huijie Leng, Jian Li, Xinyu Li, Caimei Wang, Yun Tian, Zhongjun Liu

**Affiliations:** a Department of Orthopedics, Peking University Third Hospital, Beijing, 100191, People's Republic of China; b Department of Materials Science and Engineering, College of Engineering, Peking University, Beijing, 100871, People's Republic of China; c Shanxi Key Laboratory of Biomedical Metal Materials, Northwest Institute for Non-ferrous Metal Research, Xi'an, 710016, People's Republic of China; d Beijing AKEC Medical Company Ltd., Beijing, 102200, People's Republic of China

**Keywords:** Three-dimensional-printed porous implants, Large bone defect treatment, “Implant-bone” interface fusion, Osseointegration, 3D, three-dimensional, BVF, bone volume fraction, CAD, computer-aided design, EBM, electron beam melting, FEA, finite element analysis, micro-CT, micro-computed tomography

## Abstract

Bone defect repairs are based on bone graft fusion or replacement. Current large bone defect treatments are inadequate and lack of reliable technology. Therefore, we aimed to investigate a simple technique using three-dimensional (3D)-printed individualized porous implants without any bone grafts, osteoinductive agents, or surface biofunctionalization to treat large bone defects, and systematically study its long-term therapeutic effects and osseointegration characteristics. Twenty-six patients with large bone defects caused by tumor, infection, or trauma received treatment with individualized porous implants; among them, three typical cases underwent a detailed study. Additionally, a large segmental femur defect sheep model was used to study the osseointegration characteristics. Immediate and long-term biomechanical stability was achieved, and the animal study revealed that the bone grew into the pores with gradual remodeling, resulting in a long-term mechanically stable implant-bone complex. Advantages of 3D-printed microporous implants for the repair of bone defects included 1) that the stabilization devices were immediately designed and constructed to achieve early postoperative mobility, and 2) that osseointegration between the host bone and implants was achieved without bone grafting. Our osseointegration method, in which the “implant-bone” interface fusion concept was used instead of “bone-bone” fusion, subverts the traditional idea of osseointegration.

## Highlights

• A new “implant-bone” interface fusion concept for large bone defect treatment was realized using 3D-printed porous implants.
• Osseointegration was achieved without bone grafting.
• An animal study revealed that the bone grew into the pores with gradual remodeling.
• Immediate and long-term biomechanical stability was achieved by this method.

## Graphical abstract

**Figure:**
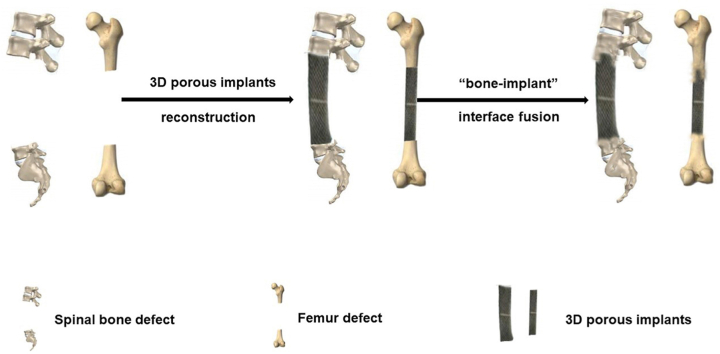

## 1 Introduction

Large bone defects caused by trauma, infection, or tumor resection [[1], [2], [3]] remain a challenging clinical issue. Approximately 5%–10% of all bone fractures are associated with delayed healing or non-union [4], whereas almost 100% of segmental bone loss fractures result in non-union [5]. Globally, over 2.2 million bone graft surgeries are performed annually to reconstruct bone defects in orthopedics, neurosurgery, and dentistry [6].

The “gold standard” large bone defect treatments include external frame fixation for bone transport during distraction (Ilizarov technique) [7,8], induction of bone regeneration via a biological membrane (Masquelet technique) [2,9], autogenous vascularized cortical bone graft [10], and titanium mesh [11]. These “gold standard” treatments are built on the idea of “bone-bone” fusion, in which the bone at both ends of the defect grows through it to integrate; however, these treatments include several disadvantages. For example, bone transport via the Ilizarov procedure requires a long time to heal, during which patients cannot resume normal activities. In addition, this procedure is unsuitable to reconstruct large spinal defects. Moreover, the Masquelet and autogenous vascularized cortical bone graft methods cannot achieve immediate stability after surgery, require large amounts of allogeneic/autogenous bone, and often require additional surgery to prevent donor site morbidity because of insufficient graft material [12,13]. Furthermore, titanium mesh requires a large amount of bone graft material, and postoperative complications, including loosening, subsidence, or displacement of the titanium mesh, often occur after surgery [14]. In addition, the Ilizarov and Masquelet techniques can not address the bone defects in the metaphysis, which exhibits an anatomical structure distinct from that of the diaphysis. The traditional “bone-bone” fusion technique has the following disadvantages in treating large bone defects: inability to withstand mechanical challenges at the load-bearing sites immediately after surgery [15], and a long treatment process. Although some alternative scaffold-based strategies have been evaluated to reconstruct bone defects [[16], [17], [18]], no reliable solution has been identified to treat large bone defects. Furthermore, most of these methods require allogeneic/autogenous bone filling [19]. 3D-printed porous Ti alloy implant, Ti6Al4V, was developed with demonstrated advantages in reconstructing bone defects, including an accurate shape and size with no need for bone grafting and realize immediate stabilization allows for early post-operative off-bed mobility, along with its porous feature that favors bone ingrowth. Although 3D-printed porous implants have been used to repair bone defects, little is known about the long-term therapeutic effects and osseointegration characteristics of the use of 3D-printed porous titanium implants without an allogeneic or autogenous bone graft to treat large bone defects.

To circumvent the drawbacks of the traditional “bone-bone” fusion methods for the treatment of large bone defects, we proposed a new concept and approach for large bone defect reconstruction, termed “implant-bone” interface fusion, in which the bone at both ends of the defect grows into the pores of the implant to achieve implant-bone fusion. It should be mentioned here that If the bone at both ends of the defect site grow through the defect to fuse with each other, it is called “bone-bone fusion”. If the bone adjacent to both ends of the porous implants just grow into and fuse with the porous implants rather than meet each other, it is called “implant-bone interface fusion”. To achieve immediate biomechanical stability and long-term “implant-bone” fusion, this technique comprises a one-stage implementation of the three-dimensional (3D)-printed porous titanium implant, which is custom-made by an electron beam melting (EBM) technique [20].

We employed the “implant-bone” interface fusion method to reconstruct patient-specific large bone defects that had different underlying causes. Postoperative follow-up was performed to monitor long-term outcomes. Further, we used a 4-cm segmental femur defect sheep model to study the osseointegration characteristics of the implants.

## 2 Materials and methods

### 2.1 Implant design and fabrication

A pore size of 400–600 μm, strut diameter of 240–320 μm and porosity of 60%–80% was adopted for the 3D-printed porous titanium implants in this study because previous study demonstrated that the pore size at this range is beneficial for in-growth of bone and vessels [21]. The size and shape of the implants were designed according to the 4-cm segmental femur defect sheep model. The critical-size bone defect of the experimental animal model was defined by Schmitz as the defects of a size that will not heal during the lifetime of the animal [22]. And in clinical, the defects whose length exceed 50% of the bone circumference or 2 cm was always referred to as critical-size bone defect [23]. Hence, we define 4 cm bone defect in the sheep femur of present study as the large-bone defect. To achieve immediate stabilization for early post-operative mobility and favorable bone healing environment, a screw-plate system was designed to unite with the porous implant as one (S1 Appendix Fig. S4). And to circumvent the effect of the fixing screws on the objective results, we remove all fixing screws before high-resolution micro-CT scanning and biomechanical test. For implant fabrication, the 3D structure was first projected using Mimics software; the acquired data were then entered into the EBM S12 system (Acram AB, Sweden). This system could melt the titanium powder (Ti6Al4V) and remold the implant according to the computer-aided design (CAD) model. Finally, the implants were air-blasted and cleaned ultrasonically to remove excess particles and pollutants.

### 2.2 Experimental animal handling

Based on the sample size calculation, using formula N = ((Z1-α/2+Z1-β)2σ2(1 + 1/k))/δ2 (where δ is the standardized mean difference, σ is the standard deviation, α = 0.05, β = 0.1 and k = n1/n2), a sample size of two for each group was determined as appropriate. However, to account for potential sample degeneracy, we used a sample size of four per group. Hence, 12 non-GMO, specifically pathogen-free, healthy, mature male Small Tail Han sheep (weight, 47.8 ± 5.3 kg; age, 17 ± 2.9 months) underwent a 4-cm segmental femur defect osteotomy. Based on the breeding time, all sheep were randomly and equally assigned to one of the three groups, namely, the 1-, 3-, and 6-month groups, using a two-by-two test matrix. The group names represented the time that each defect was allowed to heal after surgery.

All sheep were quarantined according to the Beijing standard for experimental sheep. The study animals were bred at the Department of Laboratory Animal Science at Peking University Health Science Center and were cared for according to the principles of the Guide for the Care and Use of Laboratory Animals, after obtaining approval from the Animal Ethics Committee of Peking University Health Science Center (approval no. LA2014214).

### 2.3 Surgical procedures

For the critical-size (4 cm) osteotomy, a lateral approach to the right femur was selected. The sheep were placed in the lateral decubitus position and administered induction analgesia using propofol (4–8 mg/kg intravenous injection). Anesthesia was maintained with 1.0%–2.0% isoflurane in oxygen. Penicillin, 1 g intravenous, was administered prophylactically just before and at the end of surgery. Parecoxib sodium (40 mg intravenous injection) was administered for postoperative analgesia.

The area over the right femur was aseptically prepared and incised 15 cm longitudinally using a scalpel. The femur was separated from the attaching muscles using a periosteal detacher through the intramuscular spaces. Bleeding was stopped with using electric cautery. A 4-cm cylindrical bone fragment, together with the periosteum, was excised in the mid-diaphyseal tibia with an oscillating saw (Stryker 4207, Kalamazoo, Michigan) using a custom-made sawing template. The implant was then fixed in the proximal and distal bone fragment to reconstruct the 4-cm segmental femur defect, using a screw-plate system (S1 Appendix Fig. S4). The wound was carefully cleaned and rinsed with saline solution. The subcutaneous tissue and skin were then closed with continuous sutures. Finally, in all sheep, the wound was covered with sterile gauze and synthetic cotton. After surgery, analgesics (Parecoxib, 80 mg/day) were administered and the animals were allowed to move freely.

### 2.4 Radiographic analyses

Mineralized callus formation and bridging of the critical-size osteotomy gap were evaluated immediately after sacrifice (via an anesthetic overdose) by employing radiological and axial spiral computed tomography (CT; Siemens, SOMATOM Definition Flash 64 Munich, Germany) with the following parameters: X-ray source current, 200 mA; voltage, 120 kV; 21-cm field of view, and 3-mm slice thickness. To perform radiography, intravenous injections of pentobarbital sodium (30 mg/kg) were administered for short-term sedation. Bridging of the critical-size osteotomy gap was scored according to the bony bridging length along the surface of the implants. A score of 1 indicated that bony bridging occurred along less than one-third of the implant surface. A score of 2 indicated that bony bridging occurred along less than two-thirds of the implant surface. A score of 3 indicated that complete bony bridging occurred along the implant surface.

### 2.5 Micro-CT analysis

The experimenter who conducted the micro-CT analysis was blinded to all assessed groups. After the fixed screw was removed, high-resolution (20 μm) micro-CT was performed, using an Inveon MM system (Siemens, Munich, Germany), to measure the amount and distribution of bone in the mid-diaphyseal section of the femur that contained the defect. Each specimen was analyzed using segmentation software. The region of interest included the bone within the implant plus the bone proximal to it, whose boundary was manually positioned for definition. The 3D reconstructions were produced from two-dimensional images using a 3D visualization system (Inveon Research Workplace, Siemens, Munich, Germany). The ratio of the total amount of the bone region was calculated as the bone volume fraction (BVF), determined as the bone/tissue volume ratio. The in-grown new bone was distinguished from soft tissue and metal implant by partition of different Hounsfield units (HU). Newly formed bone was identified from the implant material by adjusting the threshold value (1000HU–3885HU). Two regions of interest (ROIs) were determined in the workstation to characterize the newly formed peri-implant and intraporous bone. The former include the peri-implant region at the peripheral 2 mm around the implant and the later is the intraporous region within the porous implant. The peri-implant bone fraction was defined as the ratio of bone volume to the total volume of the region, while the intraporous bone fraction was the ratio of bone volume to the total volume of the pores.

### 2.6 Three‐point bending test

After sacrifice, the femurs of each animal were explanted. The fixation screws were carefully removed from the operated femur, which then underwent preparation for the three‐point bending test that was performed using a hydraulic material testing system (Landmark, MTS Systems Co., Minneapolis, MN, USA) with a 5-kN load cell. The load was applied to the midpoint of the unsupported length of the femur at a rate of 3 mm/min. The ends of the tested specimens were not fixed. The two lower supports were separated by a 13-cm distance, and the upper loading point was opposite to the fixation side. For comparison, the yield strength of the sheep's non-operated femur was also tested and served as the blank group.

### 2.7 Finite element analysis

A finite element analysis (FEA) established an FEA model of the 4-cm segmental sheep femur defect that was reconstructed by the 3D-printed individualized porous implant under the three-point bending test and further analyzed the stress distribution and clinical safety of the implant at 3 months after surgery. The finite element simulative analysis model of the implant-bone complex was established using CT data and was generated using Mimics Research 20.0 software (Materialise, Belgium). The simulation model components included the sheep femur, porous scaffold, and fixing plate. The material attributes are listed in S1 Appendix, Table S1. The model was imported into ABAQUS (6.14) software for establishment and calculation. A 1000-, 2000-, and 3000-N load was applied to the midpoint of the scaffold to simulate the working conditions of the three-point bending test. The displacement and stress distributions of the implant-bone complex were calculated to verify the FEA validity and peak stress of the implant-bone complex. Linear-regression was used for a difference analysis of the implant-bone complex mechanical response (displacement) between the finite element model and the actual specimen in the three-point bending test. The regression coefficient (R2 = 0.96 > 0.82) was calculated by inputting the displacement value in SPSS statistical software version 16.0 to demonstrate the validity of the present FEA (S1 Appendix, Fig. S5) [24].

### 2.8 Fluorescent labeling analysis

In vivo sequential fluorescent labeling was performed to label the newly formed bone at different time points and to determine the osseointegration efficiency at different stages and the osseointegration direction (S1 Appendix, Table S2). Specifically, calcein green (10 mg/kg, Sigma, St. Louis, MO, USA) and tetracycline (20 mg/kg, Sigma) were injected intravenously in succession after implantation. The implant plus the adjacent bone were harvested, fixed in 4% paraformaldehyde for 1 week, and dehydrated in a graded ethanol series (50%, 70%, 80%, 90%, 95%, and 100% ethanol) under vacuum conditions for 4 days each. The samples were embedded in polymethyl methacrylate (PMMA, Technovit 7200 VLC) and sliced into thin sections (200–300 μm) in the mid-sagittal plane to obtain central views of the implant and the adjacent bone. The sections were polished to a thickness of 100–150 μm using a polishing machine (Exact band saw; Exact Apparatebau, Norderstedt, Germany). A laser beam at wavelengths of 488 nm and 405 nm sequentially excited the calcein green and tetracycline fluorescence, respectively. A fluorescence microscope (Leica, Germany) was used to observe the newly formed calcein- and tetracycline-labeled bone.

### 2.9 Histologic analysis

**Fig. 1:**
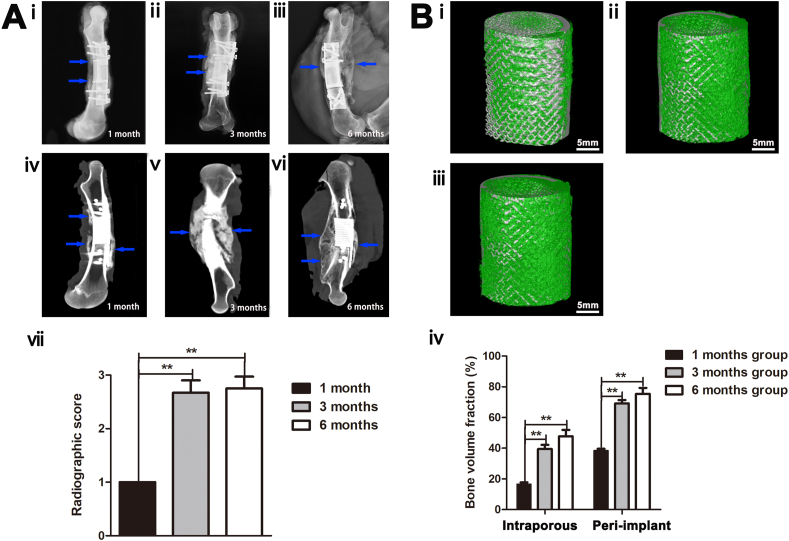
Radiological and biomechanical analyses of the 4-cm femur defect reconstruction with three-dimensional-printed porous Ti6A14V implants. (**A**) (i-iii) Radiographs at 1, 3, and 6 months after implantation, respectively. (iv-vi) Computed tomography images at 1, 3, and 6 months after implantation, respectively. Blue arrows denote newly formed bone in the defect sites or on the implant's outer surface. (vii) Radiographic scores of the groups (n = 4). (**B**) (i-iii) Three-dimensional reconstruction images of the 1-, 3-, and 6-month groups after sacrifice, respectively (gray denotes titanium alloy, and green represents newly formed bone). (ⅳ) Quantitative results of the bone volume fractions in the peri-implant and intraporous regions of the implants in each group (n = 4).

Following the fluorescent labeling assessment, the 100-150-μm slices underwent Goldner trichrome staining and were digitally imaged using a panoramic scanner (Nano Zoomer, Hamamatsu) to capture images of the whole section (Fig. 1). Goldner trichrome stained the implant black and the cartilage purple. Osteoid, a sign of active direct bone formation, was stained orange-red and mature mineralized bone was stained green. The composition and distribution of the newly formed bone were evaluated using the BIOQUANT Image Analysis System (BIOQUANT Image AnalysiCorp., Nashville, TN, USA). The available pore space of each section was normalized to 100%, and the percentage of bone in-growth was calculated for each section. Further, to quantify the maturity of the bone in-growth of the entire specimen, the proportions of osteoid and mature mineralized bone were calculated. The bone regeneration process within the defect was examined according to the distribution of osteoid and mature mineralized bone.

### 2.10 Large bone defect treatment with individualized porous implants in patients

The study was conducted in accordance with the principles of the Declaration of Helsinki. The study protocol was approved by Peking University Third Hospital Medical Science Research Ethics Committee (IRB00006761-2016146). All patients provided informed consent.

The inclusion criteria were a bone defect length of at least 4-cm or two spinal vertebral bodies; postoperative follow-up time >0.5 years; and no implant-associated infection. A total of 26 patients were selected, with an average follow-up duration of 28.44 ± 8.23 months. Each patient underwent treatment with a 3D-printed individualized porous implant for their large bone defect, the median length of which was 74.76 (range, 56.20, 120.57) mm. After measurement in the radiographic images, the median length of the bone penetration into the porous implant or the callus on the outer surface of the 26 bone defects was 43.96 (range, 31.92, 83.97) mm. These patients were heterogeneous with respect to the underlying cause of the defect (tumorous destruction, infection, or trauma) and the defect site (spine or limb bone). A total of 19 patients had spinal defects (5 in the cervical spine, 14 in the thoracolumbar spine), 6 patients had limb bone defects, and 1 patient had a pelvic defect. The underlying causes of the defects were tumor in 20, bone infection in 5, and trauma in 1 patient. Each patient received an individualized Ti6Al4V implant that was rapidly prototyped using the EBM S12 system (Acram AB, Sweden) on a CAD workstation based on 3D-CT data of the defect. During the entire procedure, bone grafts or osteoinductive materials were not employed. Patients received radiographic (X-ray or CT) imaging follow-up at different time points, depending on their clinical conditions. This study was registered at ClinicalTrials.gov (NCT04466397).

In this study, three typical patients who received 3D-printing implant treatment for large bone defects in the spine, pelvis, and limb bone caused by tumorous destruction and bone infection were selected and presented.

#### 2.10.1 Case 1: A 19-cm spinal defect caused by chordoma

A 40-year-old man experienced severe low back pain for 5 months due to a chordoma that extended from the 12th thoracic vertebrae (T12) to the 3rd lumbar vertebrae (L3), which led to compression of the spinal cord and nerves (S1 Appendix, Fig. S6). After a cycle of radiotherapy, we performed a T12-L3 total en bloc spondylectomy and a T7L5 pedicle screw fixation (S1 Appendix, Fig. S7). One month later, a 19-cm individualized customized Ti6Al4V implant was implanted and fixed using a pedicle screw to repair the T12-L3 defect. Radiographic follow-up images of the Ti6Al4V scaffold were performed immediately and at 1, 3, 7, 12, 24, 28, 32 and 36 months after the last surgery. The patient has provided consent for the use of his photographs in the manuscript.

#### 2.10.2 Case 2: An 11-cm femur defect caused by osteomyelitis

A 64-year-old woman sustained an open fracture of the left femur shaft due to a car accident and underwent open reduction and internal fixation at her local hospital 4 years prior. However, the fracture did not heal properly (non-union) due to osteomyelitis that occurred 1 year after surgery. Hence, antibiotic-laden bone cement was implanted after a thorough debridement at the local hospital. Unfortunately, radiographic examination conducted 7 months after the treatment indicated that non-union remained and a bone defect had formed (S1 Appendix, Fig. S8A). Her left leg motion was limited, and she experienced pain at the fracture site. She was admitted to our hospital and diagnosed with osteomyelitis of the left femur.

After a thorough debridement and sequestrectomy of the non-union area, we fixed the proximal and distal femur with an external fixator (S1 Appendix, Fig. S8B) and applied vacuum sealing drainage. During the subsequent 37 days, she underwent debridement four times. After the infection was controlled, the resulting 11-cm defect was filled with a vancomycin-laden bone cement spacer (S1 Appendix, Fig. S8B). Two months later, the patient received an individualized, patient-specific Ti6Al4V implant with an intramedullary nail for stabilization, without any autogenous or allogeneic bone use. Radiographic follow-up images of the Ti6Al4V scaffold were obtained immediately and at 2, 5, 8, 14, and 20 months after the last surgery.

#### 2.10.3 Case 3: A 7-cm pelvis defect caused by osteosarcoma

A 39-year-old woman was diagnosed with pelvic osteosarcoma. The bone defect caused by the osteosarcoma resection was reconstructed using an individualized customized Ti6Al4V implant. Unfortunately, the patient had to undergo hindquarter amputation because of a local recurrence of the osteosarcoma 18 months later. The implant-bone complex specimen was removed for micro-CT and histological analysis, the specific procedures of which were the same as the ones detailed in the “Micro-CT analysis” and “Histologic analysis” portions of the animal experiment.

## 3 Results

### 3.1 General observations

All sheep survived the surgery, healed well, and resumed load-bearing of the operated limb immediately after their return to consciousness (S1 Appendix, Fig. S1). Postoperative radiological images indicated successful reconstruction and stabilization of the defect by the porous implant, with fixation provided by nails and plates. The mean operative time was 74 min.

### 3.2 Radiographic analyses

Radiographic images documented progressive new bone formation within the defect and were used to score the healing process. The blue arrows in Fig. 1A–i, -iv indicate mineralized callus formation on the implant-bone junction surface 1 month after implantation. Three months after implantation, a thick, mineralized callus grew along the implant surface on the contralateral side of the plate and had bridged through the defect (Fig. 1A–ii, -v). The volume of bone tissue bridging the defect was greater, and the bone tissue structure appeared more regular in the 6-month group than in the 3-month group (Fig. 1 A-iii, -vi). The bone defects of all groups showed a clear spatial difference in healing, with bone growth predominantly seen on the contralateral side of the plate fixation system, and advanced bone formation on the proximal femur. The radiographic scores of the 3- and 6-month groups were significantly higher than those of the 1-month group (Fig. 1A-ⅶ). There were no significant differences in scores between the 3- and 6-month groups.

### 3.3 Micro-computed tomography analysis

To explore the osseointegration of the 3D-printed porous Ti6Al4V implants in the three groups, we quantified the bone formation both around and within the implant using micro-computed tomography (micro-CT) analysis (Fig. 1B–i, -ii, -iii). The 3D reconstruction images confirmed better osseointegration in the 3- and 6-month groups than in the 1-month group. The (BVF) of the peri-implant and intraporous regions was significantly greater in the 3- and 6-month groups than in the 1-month group (p < 0.01) (Fig. 1B–iv). There was no significant difference in BVF between the 3- and 6-month groups.

### 3.4 Three-point bending test

**Fig. 2:**
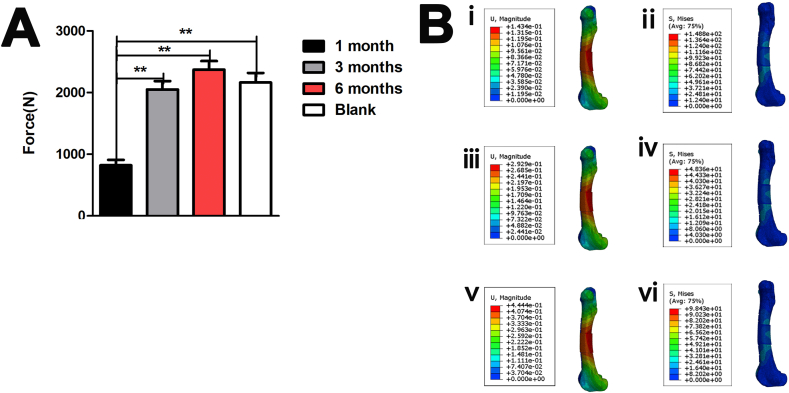
Biomechanical analysis of the 4-cm femur defect reconstruction with three-dimensional-printed porous Ti6A14V implants. (**A**) Three-point bending strength of the samples in the groups (n = 4). (**B**) Stress distribution of the implant-bone complex under (ii) 1000 N, (iv) 2000 N, and (vi) 3000 N. The displacement distribution of the implant-bone complex under (i) 1000 N, (iii) 2000 N, and (v) 3000 N **p < 0.01, *p < 0.05.

The 6-month group had the highest bending strength (2370 ± 141 N) (Fig. 2A). The bend-force resistance was significantly larger in the blank, 3-, and 6-month groups than in the 1-month group (p < 0.05), but did not significantly differ among the blank, 3-, and 6-month groups (2163 ± 156 N), indicating that the osseointegration strength could only improve to a certain extent as the growing time increased, and the biomechanical strength was insufficient until 3 months (2050 ± 135 N) after bone defect reconstruction.

### 3.5 Finite element analysis

Fig. 2. **B-ii, -iv, -vi** shows that peak stresses of the implant-bone complex are located at the edge of the bone contact with the 3D-printed Ti6Al4V implant in the 3-month group, whose for which the peak stress values were 12.09 MPa (1000 N), 3.09 MPa (2000 N), and 37.21 MPa (3000 N).

### 3.6 Fluorescent labeling analysis

**Fig. 3:**
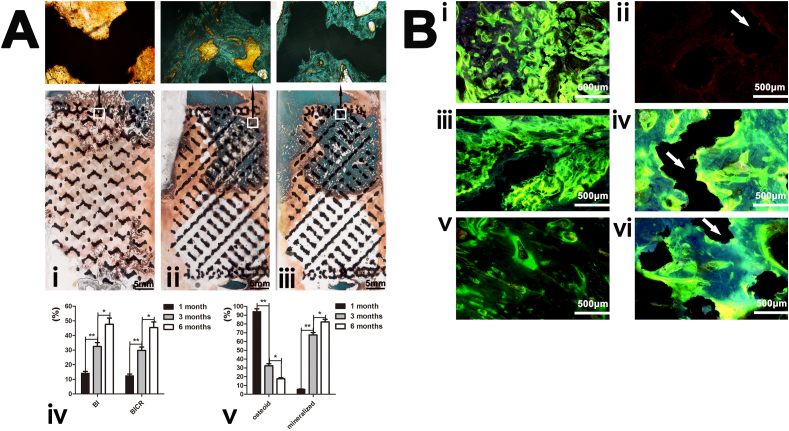
The histological analysis of the 4-cm femur defect reconstruction with three-dimensional-printed porous Ti6A14V implants. (**A**) Goldner trichrome staining (representative images) of the 1-, 3-, and 6-month groups. (iv) Quantitative results of the bone in-growth and implant-bone contact ratio of the implants in the three groups. (v) Mineralized bone and osteoid ratios of the groups (n = 10). (**B**) Fluorescence labeling (representative fluorescent micrographs) of the osseointegration around and within the pores of the implants (white arrows indicate titanium struts, and green and yellow bands denote newly formed bone indicated by calcein and tetracycline, respectively). i-vi are merged after excitation by blue and purple light. Osseointegration around the implants in the (i) 1-, (iii) 3- and (v) 6-month groups. Osseointegration within the pores of the implants in the (ii) 1-, (iv) 3-, and (vi) 6-month groups.

Fluorescence labeling revealed the osseointegration efficiency of the three groups at different sites in the defect. Comparing Fig. 3B–i, -iii, -v, it can be inferred that the osseointegration efficiency around the implants decreased with time. However, Fig. 3B–ii, -iv, -vi, demonstrate that the osseointegration efficiency inside the pores of the implants increased with time. The general osseointegration direction was from the periphery into the pores of the implants.

### 3.7 Histologic analysis

Representative histological images of the defect revealed achievement of good integration with the host bone at the proximal interfaces of the defect for all groups (Fig. 3A-i**, -ii, -iii**). A small quantity of mineralized bone and osteoid formed in the proximal side of the implants in the 1-month group (Fig. 3A–i). A proximal bone defect that bridged through the pores to the middle of the implants was observed in the 3-month group. Mineralized bone was more extensive and homogeneously distributed in the peripheral and intraporous regions (Fig. 3A–ii). Six months after surgery, the mature lamellar bone became evident within the pores of the implants on the proximal side, interlocking tightly with the titanium struts and forming continuous structures (Fig. 3A–iii).

Bone grew from the outer surface of the implants into the pores and matured gradually, indicating that the bone that bridged across the defect was in an ongoing growth process induced by the implant. Quantitative measurements of the mineralized bone, osteoid, and bone in-growth in the regions of interest confirmed the histomorphological findings. Fig. 3A shows a significantly higher bone in-growth and implant-bone contact ratio at the implants in the 3- and 6-month groups than in the 1-month group (**p < 0.01). The mineralized bone ratio was significantly higher in the 6-month group than in the 1-month (**p < 0.01) and 3-month (*p < 0.05) groups. The osteoid bone ratio was significantly higher in the 1-month group than in the 3- and 6-month groups (**p < 0.01).

### 3.8 Three representative patients

#### 3.8.1 Case 1: A 19-cm spinal defect caused by a chordoma

**Fig. 4:**
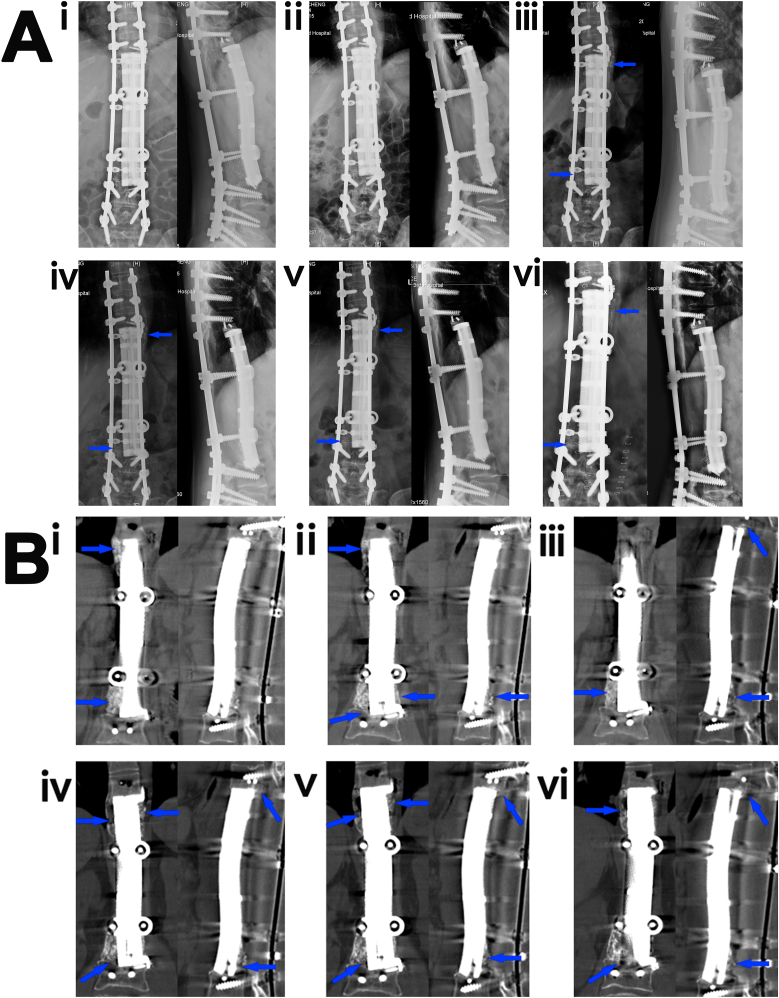
A spine defect reconstructed by a three-dimensional (3D)-printed porous Ti6Al4V implant (Case 1). (**A**) (i-vi) Radiographs of the reconstructed 19-cm spine defect at 1 (i), 3 (ii), 7 (iii), 12 (iv), 24 (v), and 32 (vi) months after implantation. Blue arrows indicate the newly formed bone in the implant-bone interface or on the outer surface of the implant. (**B**) Computed tomography images of the reconstructed 19-cm spine defect at 3 (i), 7 (ii), 12 (iii), 28 (ⅳ), 32 (v), and 36 (vi) months after implantation. Blue arrows indicate the newly formed bone in the implant-bone interface or on the outer surface of the implant.

One month after the last surgery, the patient was able to walk independently with full weight-bearing (S1 Appendix, Fig. S2A). The process of bone formation on the scaffold surface is shown in Fig. 4A and B. An obvious mineralized callus was present at the interface between the scaffold and the T9 and L3 vertebral bodies 3 months after surgery (Fig. 4B–i, blue arrows). The callus thickened and mineralized as the bone grew (Fig. 4A–iii, -vi, and Fig. 4B–ii, -vi, blue arrows). During the follow-up period, no instability of the implant-bone complex, such as loosening, subsidence, or displacement of the implant, or other mechanical complications were observed. The patient is currently enjoying a high postoperative quality of life (S1 Appendix, Fig. S2).

#### 3.8.2 Case 2: An 11-cm femur defect caused by osteomyelitis

**Fig. 5:**
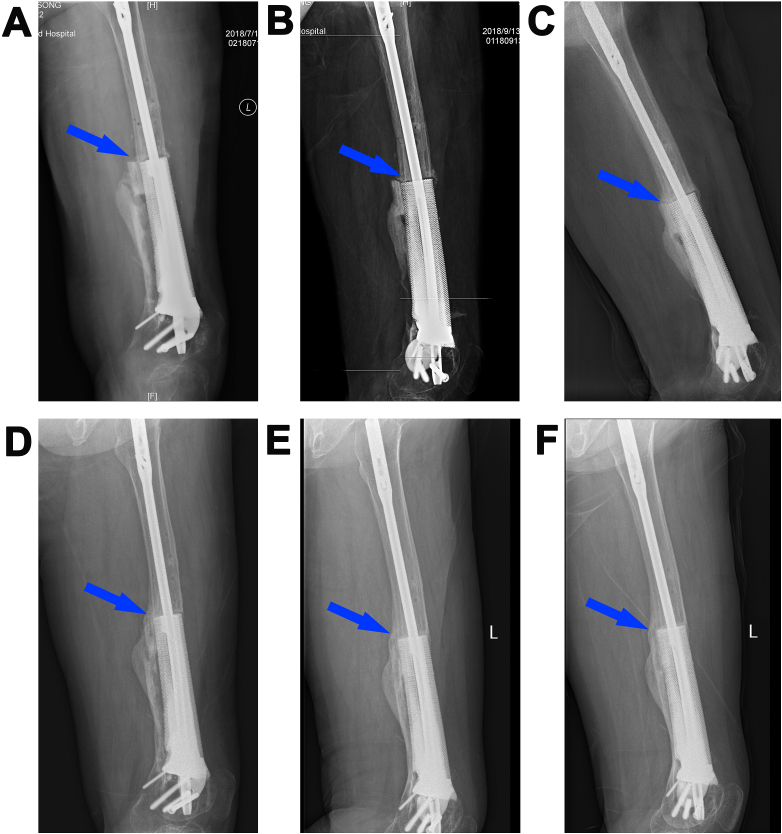
A femur defect reconstructed by a three-dimensional (3D)-printed porous Ti6Al4V implant (Case 2). Radiographs of the reconstructed 11-cm femur defect after the last surgery (**A**), and at 2 (**B**), 5 (**C**), 8 (**D**), 14 (**E**), and 20 (**F**) months after implantation. Blue arrows indicate the osseointegration between the scaffold and host bone.

One month after the last surgery, the patient could walk with full weight-bearing. The process of osseointegration between the implant and the host bone is shown in Fig. 5 (A-F). A callus was present at the interface between the scaffold and distal femur 8 months after the scaffold implantation surgery (Fig. 5D, blue arrow). A thick mineralized callus had formed and completely bridged the large bone defect on the outer surface of the scaffold 20 months after the last surgery (Fig. 5F, blue arrow). During the 20 months of recovery time, no limitations of walking or prosthetic mechanical complications were observed. No loosening or fracture of the implant was observed, and no callus absorption occurred.

#### 3.8.3 Case 3: A 7-cm defect caused by an osteosarcoma

**Fig. 6:**
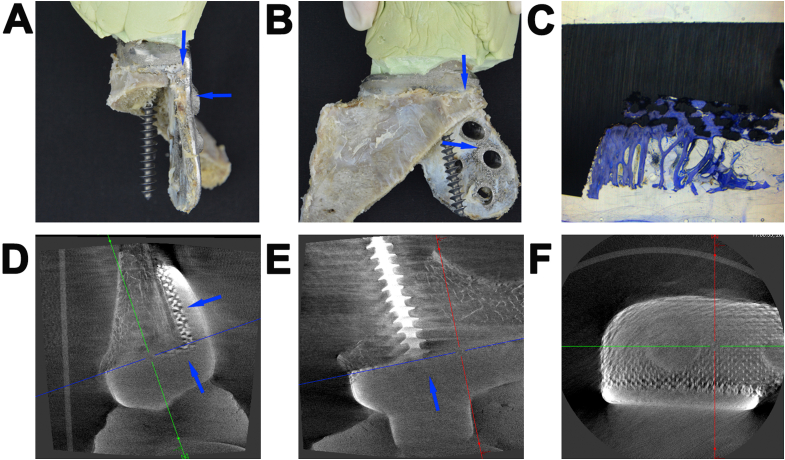
A femur defect reconstructed by a three-dimensional (3D)-printed porous Ti6Al4V implant (Case 3). Photographs of the implant-bone complex specimen from the (**A**) Lateral and (**B**) Anteroposterior views. Blue arrows indicate the site of the implant-bone contact area. (**C**) Histological images of the implant-boneimplant-bone interface, showing a high degree of new bone growth into the porous network. Micro-computed tomography images of the implant-bone contact area on the (**D**) Median sagittal, (**E**) Coronal and (**F**) Transverse sections.

Micro-CT and histological analyses were performed at the site of the implant-bone contact area (Fig. 6, blue arrows). Histological analysis revealed a high bone growth ratio (87% ± 0.78) into the porous network (Fig. 6C). Micro-CT analysis revealed that the porous implant achieved good integration with the host bone at the interface of the proximal defect (Fig. 6D and E, blue arrows), with bone trabeculae extending toward the distal part.

## 4 Discussion

**Fig. 7:**
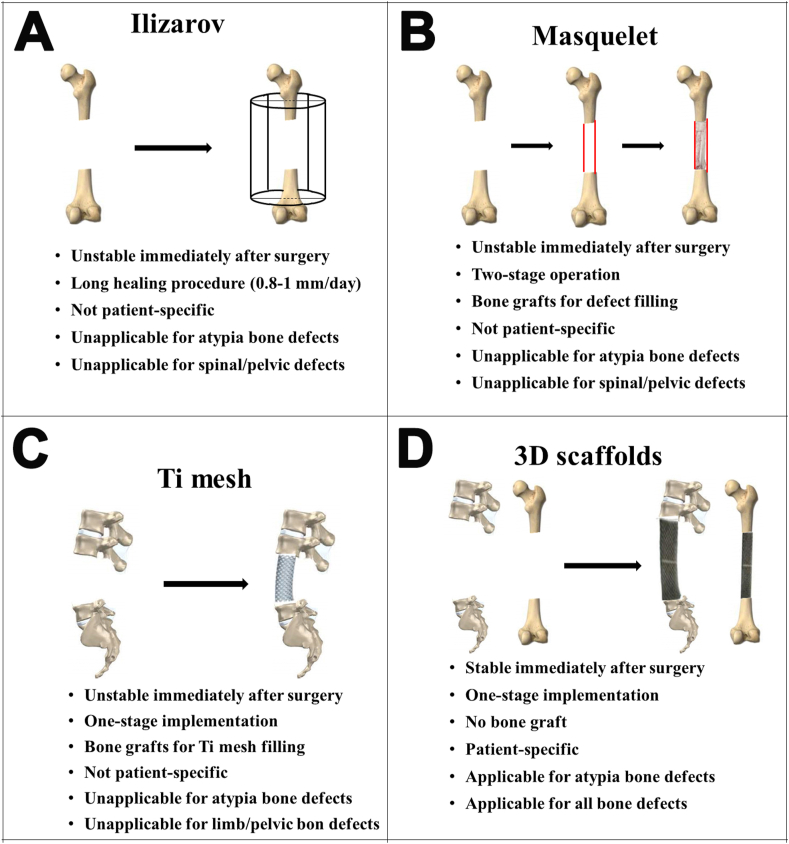
Schematic diagram and characteristic comparison of the implantation methods for the treatment of large bone defects: (**A**) Ilizarov, (**B**) Masquelet, (**C**) Titanium mesh, and (**D**) Our “implant-bone” interface fusion method.

In this study, to precisely reconstruct patient-specific large bone defects caused by tumors, infections, or trauma, we proposed a treatment strategy comprising a one-stage implementation of individualized porous implants in the absence of allogeneic/autogenous bone grafting, based on “implant-bone” interface fusion, instead of “bone-bone” interface fusion [[25], [26], [27]]. Our postoperative results indicated that the individualized porous implants had sufficient mechanical integrity to maintain the immediate stability of the defect site. At an average of 28.44 months, long-term biomechanical stability of the implant-bone complex was achieved, with extensive bone formation and reinforcing osseointegration in all patients. Fig. 7 provides a comparison of our “implant-bone” interface fusion method with other “bone-bone” fusion methods to treat large bone defects. After examining the radiographic data of 26 patients with various bone defects, we found that the “implant-bone” interface fusion occurred by bone growing through the porous implants or by a mineralized callus that bridged the outer surface of the porous implants (S1 Appendix. Fig. S3). In addition to postoperative immediate biomechanical stability, the animal experiment revealed that the implant-bone complex improved the mechanical stability. Most importantly, histological analyses revealed that the bone grew into the pores of the implants and gradually matured to achieve “implant-bone” interface permanent fusion, rather than growth through the porous implants for complete “bone-bone” fusion.

The three-point bending test is commonly used to evaluate the mechanical properties of long bones. This test assesses the bending fracture load and stiffness, ultimate stress, and elastic modulus of the specimens. In this study, we examined the ultimate fracture load of the implant-bone junction to determine the osseointegration strength in the three groups. We found that there was sufficient biomechanical strength 3 months after implantation of the porous implant. Notably, FEA of the three-point bending test revealed that the peak stress of the implant-bone complex was located at the edge of the bone that was in contact with the implant. These results further verify the safety of using 3D-printed porous titanium implants for the reconstruction of large bone defects.

We found that a gradual transition from osteoid to the mineralized bone, with increased bone bridging and infiltration distance into the implant interior pores from the peripheral site, occurred in all groups, indicating that the newly formed bone could remodel and progressively mature. The white parts at the bottom of Fig. 3. (i-iii) images is the intraporous region that with no bone ingrowth. The histologic analysis demonstrated that osteogenesis started from the proximal femur implant-boneimplant-bone interface and infiltrated the distal part of the scaffold under axial stress conduction stimulation [28,29]. Our histological images also revealed that reliable osseointegration was achievable through bone growth into the interconnected pores to a certain extent, achieving a “implant-bone” interface fusion rather than extending completely through the scaffold. In our animal experiment, at most, half of the length of the scaffolds was filled with bone tissue. However, compared with the osseointegration in the 3-month group, the intrapore bone tissue in the 6-month group included more mature lamellar bone and a larger bone volume, probably because of continuous axial stress stimulation and micromotion in the implant-boneimplant-bone interface [[28], [29], [30], [31]]; gradual bone in-growth and remodeling eventually provided stability for the implant-bone complex. However, in our study, the micromotion in the implant-boneimplant-bone interface decreased and finally disappeared as the stability of the implant-bone complex increased, and bone growth in the pores halted, which explains why the bend-force resistance of the 3- and 6-month groups was not significantly different during biomechanical testing. Osteogenesis mainly exhibits a contact osteogenesis pattern, by which bone forms directly on the implant surface during the osseointegration process [32]. We found that osteogenesis mainly occurred on the porous scaffold side rather than on the fixed plate side. In addition to the stress shielding effect of the fixed plate [33,34], this phenomenon may be attributed to the porous structure being more suitable for bone formation as it circumvents the stress shielding of the solid implant; however, high porosity and interconnectivity enhances bone and vascular in-growth [35,36].

The patient in Case 2 had a typical long bone posttraumatic osteomyelitis, which is involved in approximately 10% of all open fractures and 1% of all closed fractures [37]. An accurate reconstruction of the defect by the individualized implant resulted in immediate limb stability and limb length restoration; the patient regained walking ability early after surgery. The stress stimulus caused by early load-bearing in walking promoted bone growth in the implant-boneimplant-bone interface. Bone regeneration began with the formation of the woven bone, with a less organized and mineralized structure in the early stage (Fig. 5). Newly formed bone further remodels and matures with continuous stress stimulation [[38], [39], [40]], resulting in a more stable implant-bone complex. Another critical factor for the successful osseointegration in Case 2 is the biomembrane induced by the cement spacer, which maintains a well-defined void for later placement of the porous implant and provides structural support to the complex [[41], [42], [43]]. The richly vascularized biomembrane promotes vascularity and corticalization by secreting vascular endothelial growth factor, transforming growth factor β-1, bone morphogenetic protein 2, interleukin-6, and interleukin-8 [41,42,44].

The advantages of our method over the Masquelet technique lie in the avoidance of a long healing time and autogenous bone donor site morbidity. The development of deformities has been reported with the Masquelet technique; this is due to the initiation of weight-bearing before the fracture has healed, and the nonrigid construct [45], which can be minimized by our individualized porous implant.

Although no osteogenesis promotion by the biomembrane was detected in the spinal bone defects, the postoperative radiographic results of Case 1 revealed satisfactory osseointegration at the implant-bone adjacent site. The osseointegration time differed between spinal and limb bones with porous implants. The radiographic images of Cases 1 and 2 revealed that osseointegration between the spinal bones and the implant was almost complete at 3 months after surgery, whereas that between the limb bones and the implant was almost complete at 5 months after surgery. Notably, no instability was observed at the 36-month follow-up. Our treatment of large segmental spinal defects demonstrated that the complete bridging of the bone through the porous scaffold, achieving “bone-bone” fusion, was not indispensable for successful treatment. The feasibility of this key idea was emphasized by the results of the histological and micro-CT analyses shown for Case 3, in which a high degree of new bone grew into the porous network and was tightly integrated. The satisfactory bone fusion at the implant-bone adjacent site, which is stimulated by continuous stress postoperatively (38–40), also maintains the long-term stability of the implant-bone complex. Long-term follow-up results of all patients in our study revealed that firm “implant-bone” fusion was obtained in the implant-boneimplant-bone interface. And we did not use any autogenous/allogeneic bone grafts, additional growth factors, or osteoinductive agents with the individualized porous implants, thus saving costs and avoiding autologous/allogeneic bone-related complications. Certainly, the absence of bony bridge connection in this study may lie in that the observation time is not long enough, the porous structure design of the porous implant, or the absence of any bone graft material and growth factors. But the study proved it to some extent that complete bony bridge connection is not an indispensable condition for permanent biomechanical stability or permanent reconstruction. “Implant-bone interface fusion” may also achieve the therapeutic purpose, after all, the complication of “implant-bone complex” unstability does not occur in any of our patients who treated by the 3D-printed porous Ti6A14V implants till now.

Based on the clinical outcomes of all our patients treated by the 3D-printed porous implants, we found the 3D-printed porous implants are more suitable than the traditional implants for bone defect whose anatomical shape is irregular or size is large. On the whole, the application range of 3D-printed porous implants is wide, including bone defect of different sites and pathogenesis. However, some limitations are worth noting. Although the large bone defects were stably reconstructed by the individualized porous implants, local recurrence of the tumor or infection was found in some patients. Hence, in the future, we will apply anti-infection and anti-tumor functionalization technologies for the porous titanium alloy implants [[44], [45], [46], [47], [48]] to our “implant-bone” interface fusion method for better treatment of large bone defects.

Our study presents a “implant-bone” interface fusion concept for the treatment of large bone defects, which utilizes only the individualized porous implant to treat large bone defects caused by various diseases and conditions and to systematically study its long-term therapeutic effects and osseointegration characteristics in humans and animals. Our results provide compelling evidence for the clinical use of individualized porous implants to treat large bone defects and suggest that this approach is effective for the treatment of various types of large bone defects.

## 5 Conclusions

In this work, we successfully achieved the immediate and long-term biomechanical stability by the individualized porous implants without autogenous/allogeneic bone graft or any osteoinductive agents in the treatment of the large bone defects caused by varies of pathogenesis in patients. It was also revealed by the animal study that the bone can grow into the pores to a certain extent and remold gradually, resulting in a long-term mechanical stable of the implant-bone complex to a certain level. Furthermore, the study demonstrated a new “implant-bone” interface fusion concept for large bone defect treatment, differing from the classical idea of “bone-bone” fusion method.

## Significance statement

A large bone defect is a lack of bone tissue in an area where bone should normally exist. Lack of bone tissue can be caused by trauma, infection, or surgery to remove a tumor. A large bone defect was always treated using bone graft based on the traditional “bone-bone” concept, which has obvious disadvantages. We developed a three-dimensional (3D)-printed individualized porous implant for the treatment of a large bone defect based on the “implant-bone” interface fusion concept and studied its long-term effects in 26 patients and in an animal model. We found that our 3D-printed individualized porous implants can effectively treated large bone defects without the disadvantages in the methods based on the traditional “bone-bone” concept, confirming the feasibility of the new “implant-bone” interface fusion concept for large bone defect treatment.

## Data and materials availability

All data associated with this study are present in the paper or the Supplementary Materials.

## CRediT authorship contribution statement

**Teng Zhang:** Writing – original draft, designed the study, conducted the animal surgeries and most of the animal experiments, prepared the manuscript. **Qingguang Wei:** conducted the animal surgeries and most of the animal experiments. **Hua Zhou:** conducted the animal surgeries and most of the animal experimentsm. **Zehao Jing:** conducted the animal surgeries and most of the animal experimentsm. **Xiaoguang Liu:** Formal analysis, conducted the animal surgeries and most of the animal experiments, analyzed the results. **Yufeng Zheng:** Supervision, supervised the project. **Hong Cai:** Formal analysis, analyzed the results. **Feng Wei:** conducted the patients' surgeries. **Liang Jiang:** conducted the patients' surgeries. **Miao Yu:** conducted the patients' surgeries. **Yan Cheng:** analyzed the results. **Daoyang Fan:** conducted the animal surgeries and most of the animal experiments. **Wenhao Zhou:** conducted the animal surgeries and most of the animal experiments. **Xinhong Lin:** conducted the animal surgeries and most of the animal experiments. **Huijie Leng:** Formal analysis, conducted the three-point bending tests and finite element analysis. **Jian Li:** conducted the patients' surgeries. **Xinyu Li:** Formal analysis, conducted the three-point bending tests and finite element analysis. **Caimei Wang:** designed and fabricated the scaffolds. **Yun Tian:** conducted the patients' surgeries. **Zhongjun Liu:** Supervision, supervised the project, contributed to the discussion.

## Declaration of competing interest

None.
