# Supplementary material for: Three-dimensional-printed individualized porous implants: A new “implant-bone” interface fusion concept for large bone defect treatment
Source: Bioact Mater. 2021 Apr 6;6(11):3659–70. doi: 10.1016/j.bioactmat.2021.03.030 (PMC8056181; doi:10.1016/j.bioactmat.2021.03.030)
Supplement: Multimedia component 1 [file mmc1.docx]

**Supplementary Information for**

Three-dimensional-printed individualized porous implants: a new “implant-bone” interface fusion concept for large bone defect treatment

Teng Zhang, Qingguang Wei, Hua Zhou, Zehao Jing, Xiaoguang Liu, Yufeng Zheng, Hong Cai, Feng Wei, Liang Jiang, Miao Yu, Yan Cheng, Daoyang Fan, Wenhao Zhou, Xinhong Lin, Huijie Leng, Jian Li, Xinyu Li, Caimei Wang, Yun Tian, Zhongjun Liu

**Corresponding authors**: Zhongjun Liu

Email: puthztlzj@163.com;

**This PDF file includes:**

Figures S1 to S8

Tables S1 to S2


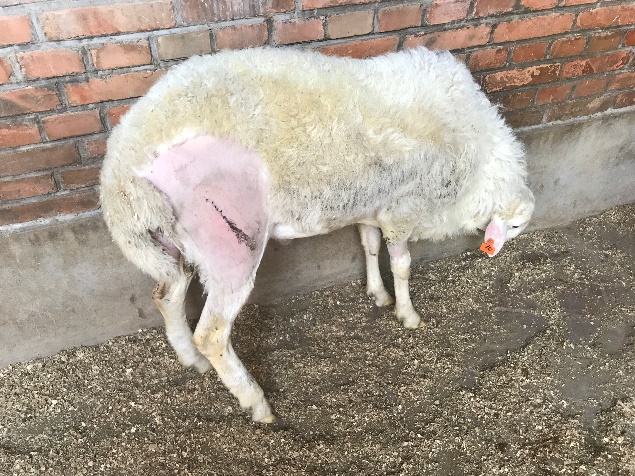


**Fig. S1.** The sheep underwent the operation and resumed load-bearing of the experimental limb immediately after gaining consciousness.


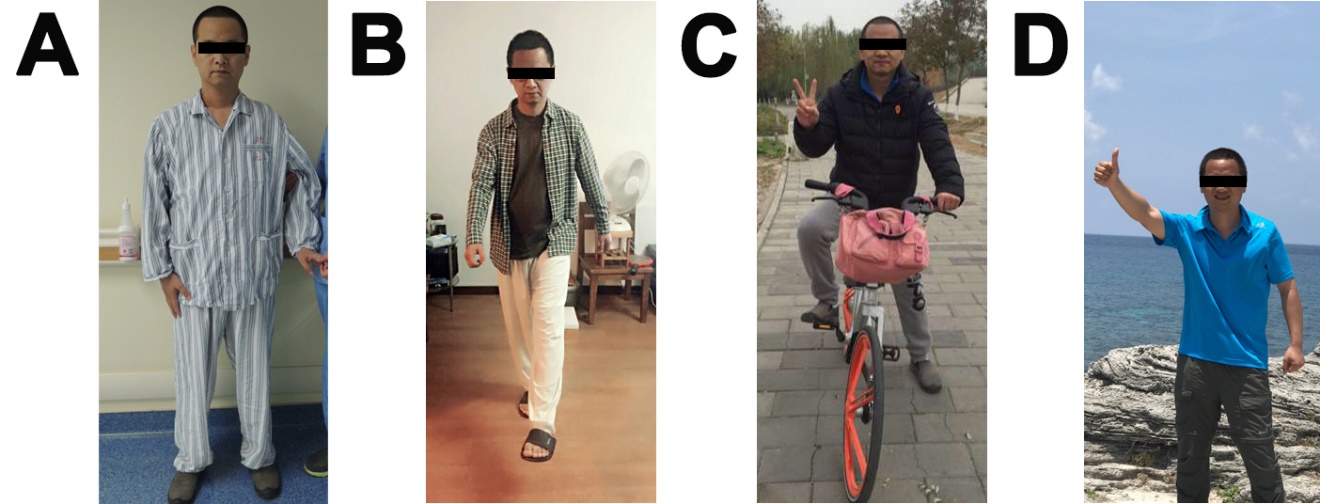


**Fig. S2.** Case 1: A 19-cm spinal defect caused by a chordoma. (A) The patient can walk with full weight-bearing and no brace one month after the implantation of the 3D-printed individualized porous implant. (B) The patient moved freely and performed activities of daily living 3 months after the implantation of the 3D-printed individualized porous implant. (C) The patient rode a bike six months after the implantation of the 3D-printed individualized porous implant. (D) The patient took a long walk while traveling 1 year after the implantation of the 3D-printed individualized porous implant.

.


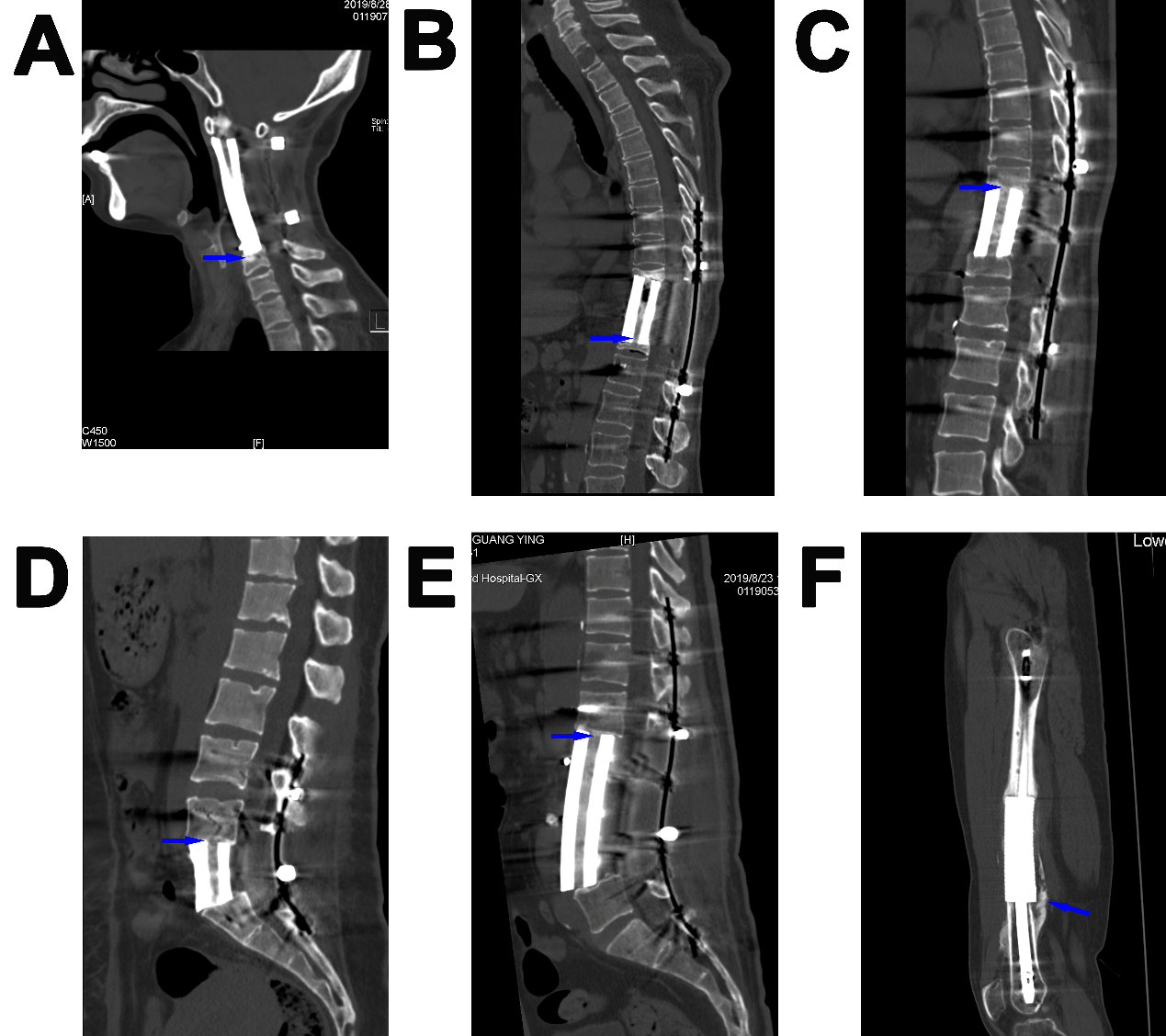


**Fig. S3.** The “implant-bone” interface fusion in patients with cervical (A), thoracic (B, C), lumbar (D, E) and femur (F) defects occurred by bone growing through the porous implants (indicated by blue arrows in A-E) or by the mineralized callus bridging at the outer surface (indicated by a blue arrow in F).


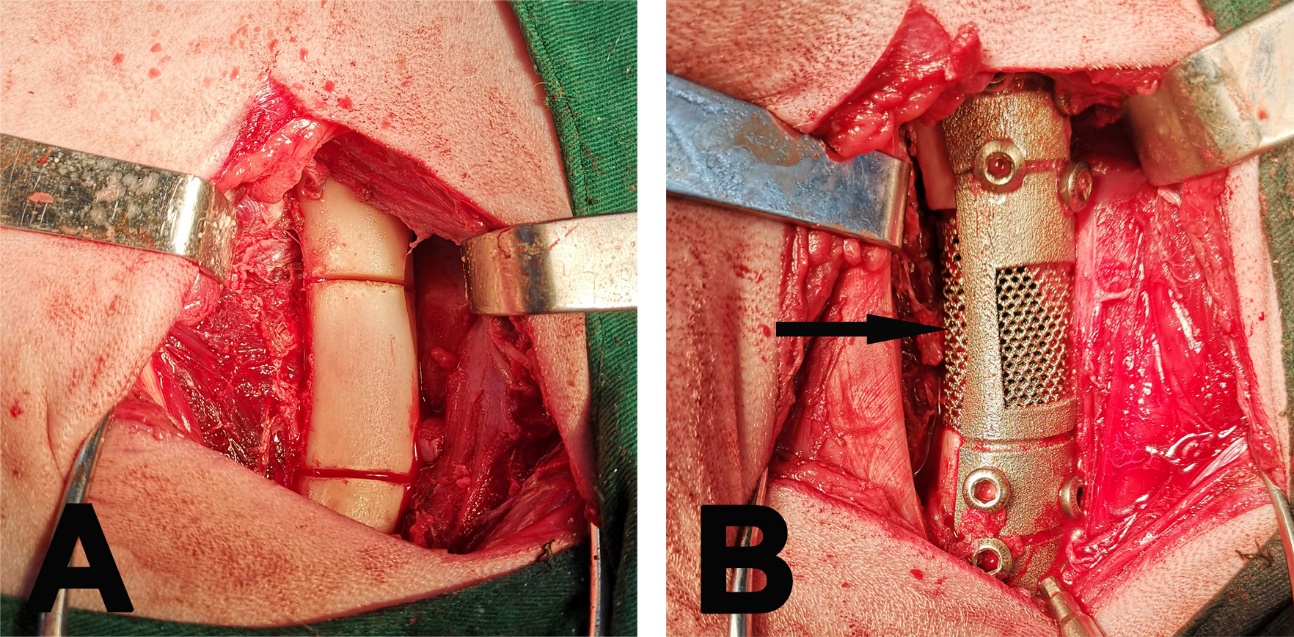


**Fig. S4.** Surgical implantation of the implant into the 4-cm segmental femur defect in the sheep. (A) The critical size (4 cm) osteotomy of the femur. (B) Implantation and fixation of the 3D-printed porous implant. The black arrow indicates the porous structure of the implants.

**Fig. S5.** Consistency between *in vitro* test results and finite element simulation results.


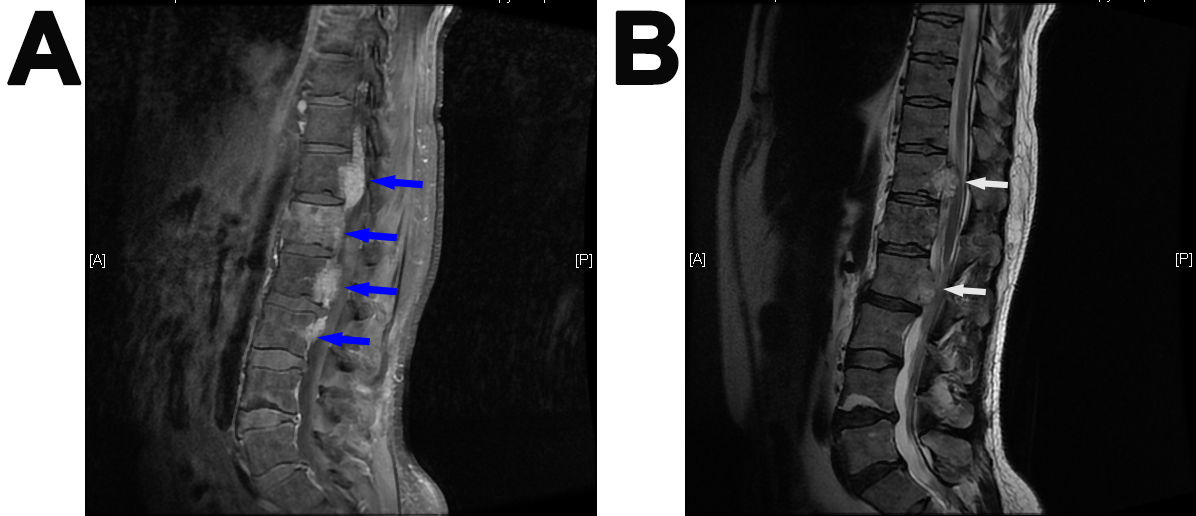


**Fig. S6.** Magnetic resonance imaging (MRI) examination of the spine before surgery in Case 1. (A) MRI (T1) image. The blue arrows indicate the chordoma that extends from the 12^th^ thoracic vertebrae (T12) to the 3^rd^ lumbar vertebrae (L3). (B) MRI (T2) image. The white arrows indicate compression of the spinal cord and nerves by the chordoma.


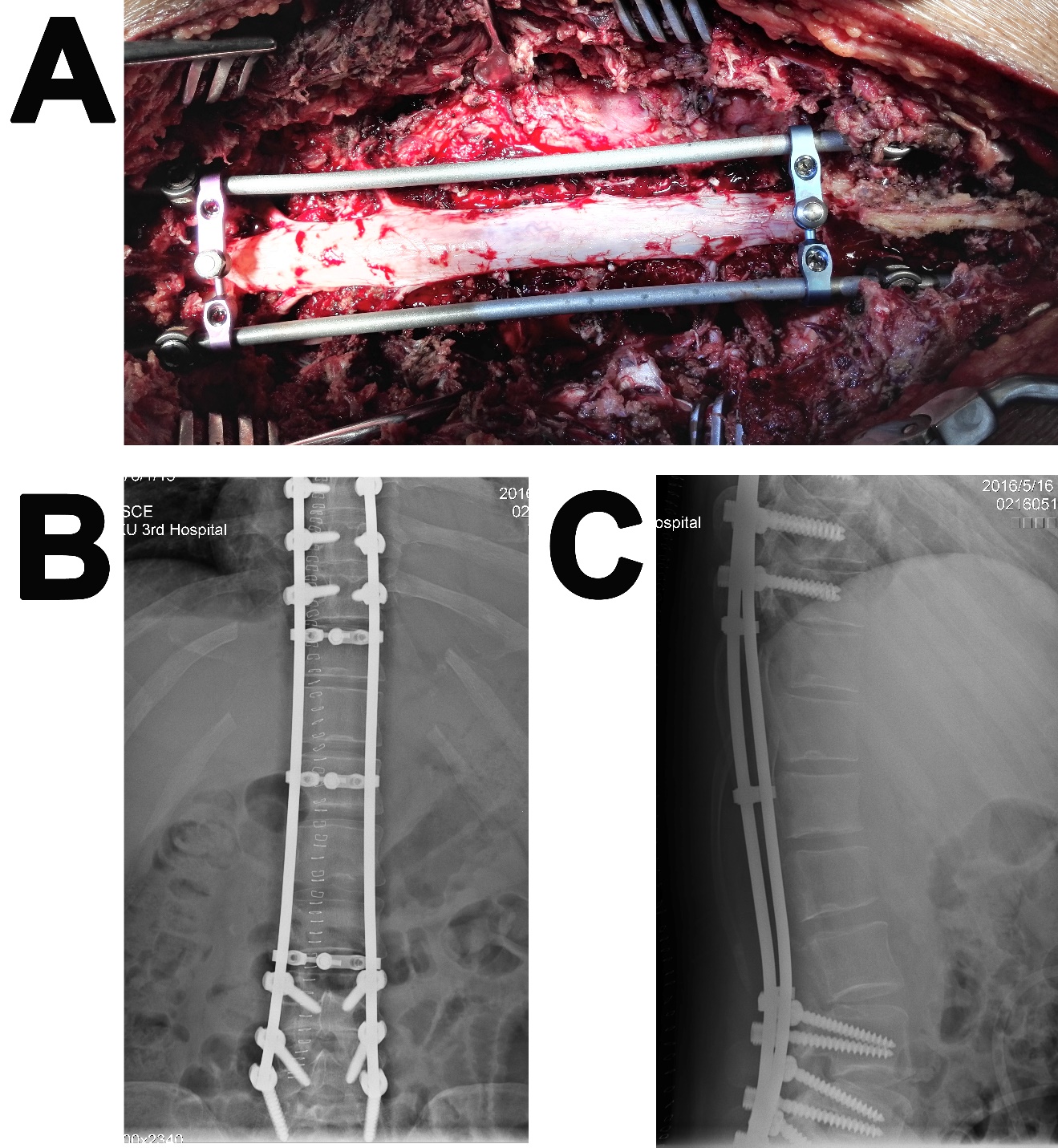


**Fig. S7.** T12-L3 total en-bloc spondylectomy and T7-L5 pedicle screw fixation of Case 1. Intraoperative photograph (A). Anteroposterior (B) and lateral (C) radiographic examination.


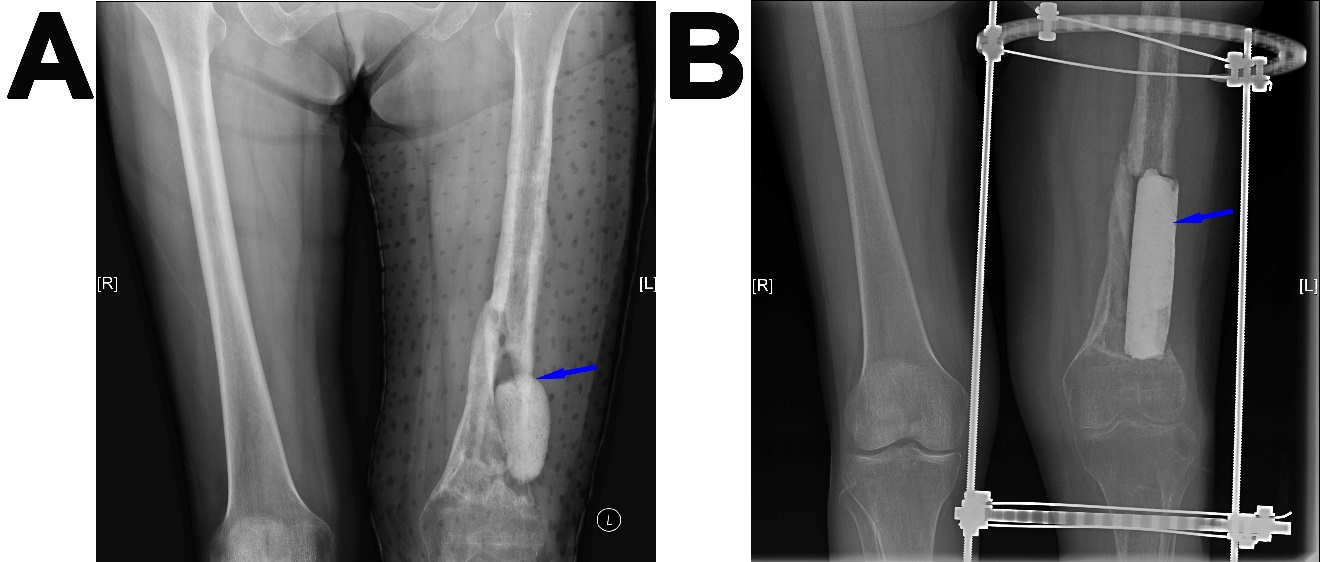


**Fig. S8.** Radiographic examination of the left femur before the 3D-printed individualized porous implant surgery in Case 2. (A) Radiographic examination of the left femur 7 months after the first surgery for Case 2. The blue arrow indicates the nonunion fracture and the femur bone defect. (B) Radiological examination of the left femur after fixation with an external fixator and filling of the defect with a vancomycin-laden bone cement spacer (as shown by the blue arrow).

**Table S1.** Material attributes

|  | Cancellous bone | Compact bone | Porous Ti6A14V implants | Solid Ti6A14V |
| --- | --- | --- | --- | --- |
| E (MPa) | 0.155 | 16.7 | 3.4 | 110 |
| μ | 0.3 | 0.3 | 0.3 | 0.3 |

**Table S2.** Fluorescent labeling time

|  |  |  | Calcein | Tetracycline |
| --- | --- | --- | --- | --- |
| 1-month group | | | 16th day | 23rd day |
| 3-month group | | | 60th day | 78th day |
| 6-month group | | | 150th day | 168th day |
